# Targeting multiple epitopes on the spike protein: a new hope for COVID-19 antibody therapy

**DOI:** 10.1038/s41392-020-00320-6

**Published:** 2020-09-19

**Authors:** Yeping Sun, Bostjan Kobe, Jianxun Qi

**Affiliations:** 1 CAS Key Laboratory of Pathogenic Microbiology and Immunology, Institute of Microbiology, Chinese Academy of Sciences, 100101 Beijing, China; 2 School of Chemistry and Molecular Biosciences, University of Queensland, Brisbane, QLD 4072 Australia; 3 Savaid Medical School, University of the Chinese Academy of Sciences, 100049 Beijing, China

**Keywords:** Immunotherapy, Infectious diseases

Lihong Liu et al.^1^ from Columbia University recently reported in *Nature* the isolation of a collection of 19 SARS-CoV-2-neutralizing monoclonal antibodies (mAbs) from five infected patients with high plasma virus-neutralizing titers. These antibodies can neutralize SARS-CoV-2 in vitro, and nine of them exhibited exquisite potency, with 50% virus-inhibitory concentrations of 9 ng/mL or less. The strategy for isolation of these antibodies included sorting SARS-CoV-2 spike protein (S)-specific memory B cells by flow cytometry and single-cell sequencing.

Currently, the global effect of the SARS-CoV-2-caused COVID-19 has continued to escalate. The disastrous pandemic has brought to the forefront the urgency and necessity for rapid development of countermeasures. Among the potential countermeasures, one promising candidate is recombinant neutralizing antibodies.

Liu et al. further mapped the epitope locations of these antibodies on the SARS-CoV-2 S by the ELISA assay. They found that some S trimer-binding antibodies bind to the receptor-binding domain (RBD) while the others bind to the N-terminal domain (NTD). A so-called “checkerboard” experiment for competition among antibodies for binding to the S trimer by ELISA grouped the non-RBD antibodies (antibodies that do not bind RBD) into four clusters (A, B, C, and D). Clusters A, B, and C are mapped to regions within or near the NTD, and the regions A and B are partially overlapping. The potent neutralizing mAbs fall exclusively into cluster A and bind to a patch on the NTD, while weaker neutralizing mAbs recognize a region at the interface between clusters A and B.

The same “checkerboard” experiment identified another four clusters among the RBD-directed mAbs (Clusters E, F, G, and H). Cluster E is a large cluster containing mAbs capable of blocking the receptor ACE2 binding; mABs of cluster G bind to an epitope on a cryptic site on the side of RBD with an “up” conformation. The most potent neutralizing mAbs group together within cluster E, suggesting that they recognize the top of RBD and neutralize the virus by competitive inhibition of receptor binding. Cluster F is likely situated between the S top and the “cryptic” site.

**Fig. 1:**
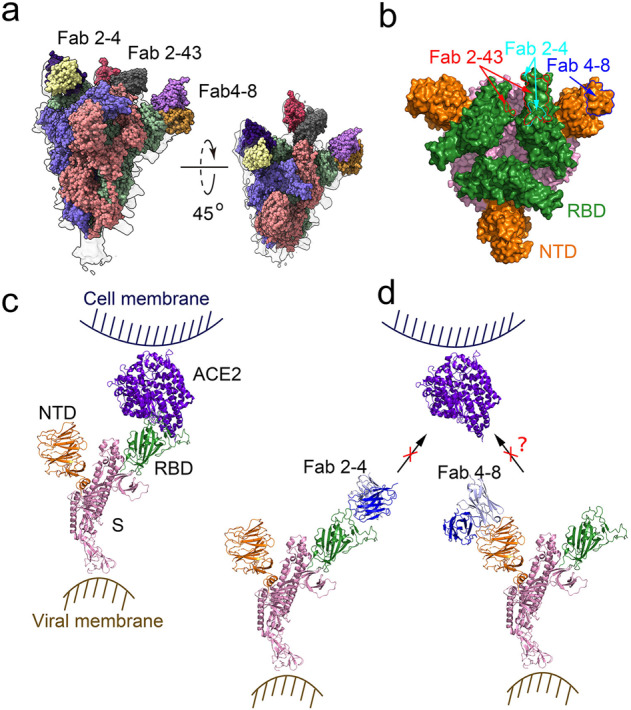
Neutralizing mAbs targeting different epitopes on SARS-CoV-2 S inhibit virus infection. **a** A structural model for SARS-CoV-2 S in complex mAbs Fab 2-4, Fab 4-8, and Fab 2-43. **b** Target regions of Fab 2-4, Fab 4-8, and Fab 2-43. **c** SARS-CoV-2 S (for clarification, only one S monomer is shown) binds to ACE2 receptor. **d** The RBD-directed Fab 2-4 inhibits virus infection via blocking SARS-CoV-2 S binding to ACE2 receptor; the NTD-directed Fab 4-8 inhibits virus infection via unknown mechanisms

The authors further solved single-particle cryo-EM (electron microscopy) structures of the S trimer in complex with several mAbs binding to different epitopes on the S protein (Fig. 1). The first corresponds to the RBD-directed mAb 2-4, which belongs to cluster E and can block ACE2 binding. The 3.2-Å resolution structures show three Fab fragments of the 2-4-mAb bind to the S trimer. Fab 2-4 binds to the spike protein near the apex and locks the RBDs in a “down” conformation, while also occluding access to ACE2. The second corresponds to the 4-8 Fab (NTD-directed, cluster A). 4-8 Fab binds to the tip of the NTDs, showing two main conformations: one for a three 4-8 Fab-bound complex of S trimer with all the RBDs in the “down” conformation (3.9 Å resolution structure), and the other for a three 4-8 Fab-bound complex of S trimer with one RBD in the “up” conformation (4.0 Å resolution structure). The third corresponds to the S trimer in complex with three 2-43 Fabs, each targeting a quaternary epitope (spanning more than one domain) on the top of RBD (5.8 Å resolution structure).

Isolation and identification of SARS-CoV-2 S-specific mAbs is now a blooming area. S-specific mAbs are being continually reported by different researchers. For example, Lv et al. reported the potent neutralizing mAb H014, which targets the side of the RBD in the open (up) conformation.^2^ Shi et al.^3^ reported that the mAb CB6 neutralizes SARS-CoV-2 by recognizing an epitope overlapping with the ACE2-binding site. Wu et al.^4^ reported a non-competing mAb pair, B38 and H4, which block RBD-ACE2 binding by targeting different epitopes on RBD. A startling highlight of Liu et al.’s report is that they successfully identified a collection of many mAbs that target multiple epitopes. Among their most potent 9 mAbs, 4 are directed to the RBD, 3 directed to the NTD, and 2 to quaternary epitopes. It is important that there are epitopes on the NTD that can be neutralizing, although the mechanisms for neutralization are unknown.

The variety of the potent neutralizing mAbs against SARS-CoV-2 reported by Liu et al. and other researchers inspires optimism that we will be able to find the highly effective and safe candidates for clinical treatment of the COVID-19. Most importantly, they provide opportunities for development of effective mAb cocktail treatments. As shown by Baum et al.^5^, the non-competing antibody cocktail was not able to induce SARS-CoV-2 S escape mutations. Therefore, the mAbs targeting multiple non-overlapping epitopes, including those discovered by Liu et al., pave a way toward developing promising mAb cocktail therapies for COVID-19.
